# Intermittent Suprasternal Neck Mass Caused by Herniation of Ectopic Thymus: Report of Two Cases

**DOI:** 10.22038/ijorl.2020.45727.2501

**Published:** 2020-11

**Authors:** Kai-Jun Tey, Bee-See Goh, Faizah Mohd-Zaki

**Affiliations:** 1 *Department of Otorhinolaryngology, Head and Neck Surgery, University Kebangsaan Malaysia Medical Centre, Jalan Yaacob Latif, Bandar Tun Razak, 56000 Cheras, Kuala Lumpur, Malaysia.*; 2 *Department of Radiology, Universiti Kebangsaan Malaysia Medical Centre, Jalan Yaacob Latif, Bandar Tun Razak, 56000 Cheras, Kuala Lumpur, Malaysia.*

**Keywords:** Ectopic, Thymus, Valsalva

## Abstract

**Introduction:**

Ectopic thymus is an uncommon cause of neck masses in children that frequently present as lateral cervical swelling especially on the right side.

**Case Report:**

We report two cases with atypical clinical presentation of ectopic thymus and superior herniation of normal thymus. Both of the patients manifested as intermittent midline mass at the suprasternal region during Valsalva manuevre. Unique ultrasound features with the location along the thymic descent together with dynamic assessment of the organ movement were essential to reach the correct diagnosis. Conservative approach was considered in these patients considering the necessity of thymus in the process of puberty.

**Conclusion:**

High index of suspicion is of utmost importance when encounter patient with similar clinical manifestation to avoid unnecessary diagnostic modalities and surgeries. Accurate diagnosis will also alleviate parents’ anxiety.

## Introduction

Neck masses are fairly common during infancy and childhood. There is a wide range of differential diagnoses of neck masses including branchial cleft cyst, thyroglossal duct cyst, cervical lymphadenopathies, thyroid and parathyroid neoplasms, aberrant thyroid, vascular tumors, ectopic thymus, benign and malignant tumours of the neck (1). However, intermittent anterior midline neck masses can be caused by several limited conditions including superior herniation of normal thymus (2), jugular phlebectasia, apical lung herniation, and laryngocoele (3).On the other hand, ectopic thymus is an infrequent etiology of cervical swelling in pediatric age group and can lead to a diagnostic dilemma. It is more commonly seen in the cervical region and presented as lateral neck mass with a preponderance to the right side (4). Ectopic thymus is rarely reported as midline neck mass which appeared with the Valsalva maneuver (5,6). We encountered two patients with similar clinical manifestation of intermittent suprasternal neck masses which become prominent during increased intrathoracic pressure. Hereby we present two cases of distinct presentation of the ectopic thymus as asymptomatic neck masses in 15 months old girl and 6-year-old boy with their management.

## Case Report

*Case* *1:*

A 15-month old girl was referred to our otorhinolaryngology clinic for the evaluation of intermittent midline suprasternal mass with the suspicion of laryngocele. The child was first noticed by her mother that she had a midline neck mass that appeared only during laughing or crying for 5 months duration. The swelling was painless and there was no precipitating factors such as previous trauma, vaccination or upper respiratory symptoms. It was not associated with compressive symptoms such as hoarseness, dysphagia or odynophagia, shortness of breath or noisy breathing. On examination, she thrived well and not in respiratory distress. 

**Fig 1:**
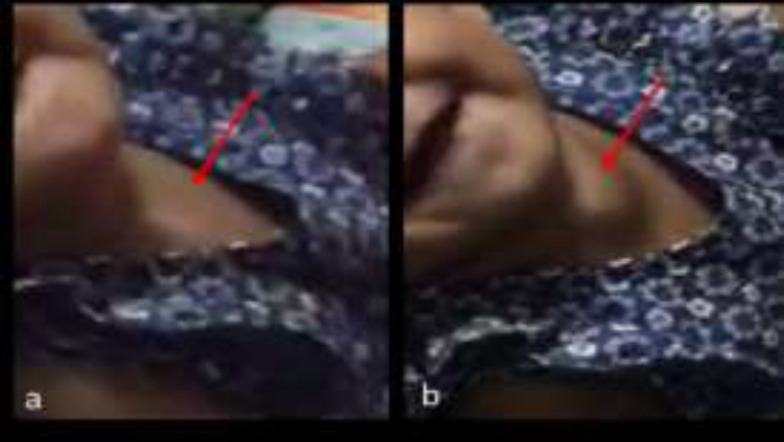
a) There is no visible neck mass over suprasternal region (as shown by red arrow) at rest. b) There is prominent neck mass over suprasternal region (as shown by red arrow) when the patient laughs

There was no abnormality detected in the neck region at rest (Fig.1a). However, a midline suprasternal swelling appeared during crying (Fig.1b). The mass was soft in consistency, 2cm x 2cm without skin changes or bluish discoloration of the skin. The mass was neither compressible nor pulsatile. 

Ultrasound neck of the patient showed the presence of ectopic thymus tissue measuring 1.2 cm (anteroposterior diameter) x 3.3cm (width) at the thoracic inlet which slides superiorly into the lower neck during crying (Fig.2a,2b).

**Fig 2a:**
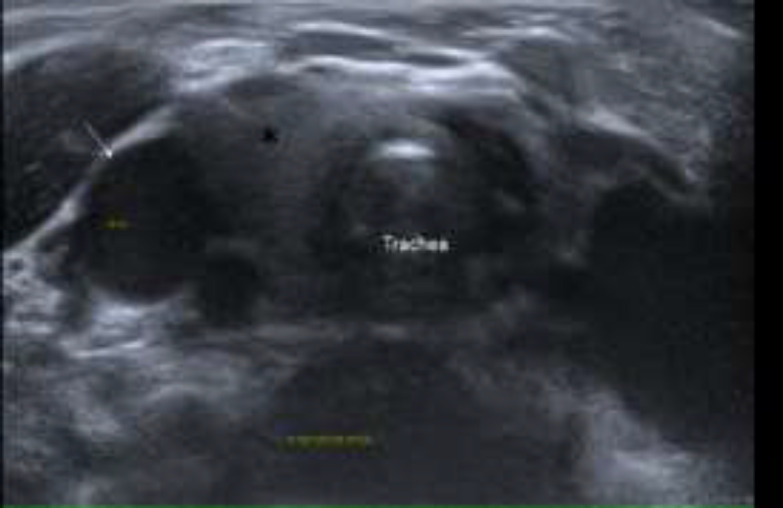
Ultrasound scan of the neck in axial view taken at suprasternal level shows sill-defined soft tissue in between right internal jugular vein (white arrow) and trachea (*)at rest

**Fig 2b:**
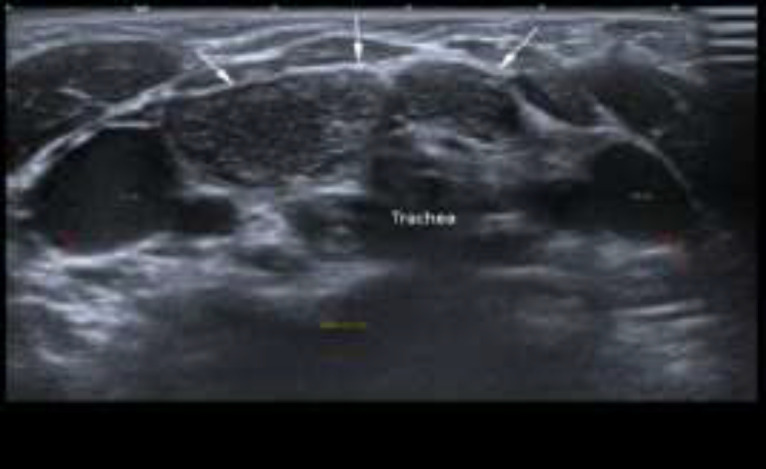
Ultrasound scan of the neck taken exactly at the same level as Figure 2a when patient was crying shows typical “starry sky appearance” suggestive of ectopic thymus (as shown by white arrows) at suprasternal region with patent bilateral internal jugular veins (as shown by red arrows).

**Fig 3:**
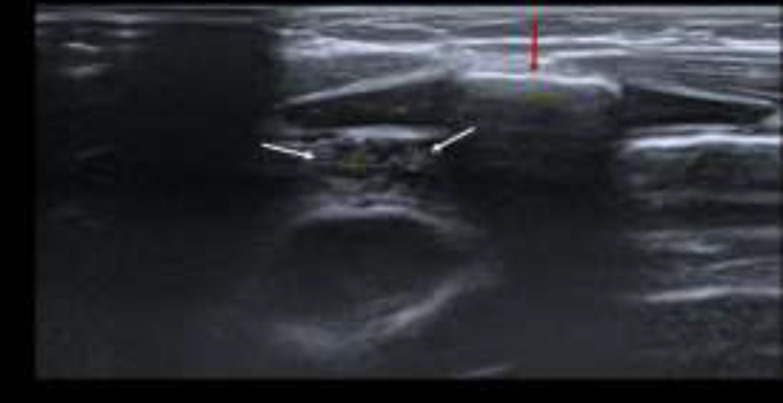
Ultrasound scan shows normal thymus (as shown by white arrows) in the thoracic inlet deep to sternum (red arrow) and no movement of the normal thymus appreciated when patient was crying

It demonstrated homogenous echogenicity and did not compress or encase the adjacent vessel such as the brachiocephalic vein, internal jugular vein, and trachea. There was no displacement of the trachea and thyroid tissue in which it indicates the soft consistency of the mass. Normal thymus tissue is also seen more inferiorly in the retrosternal region (Fig.3). 

No air-filled structures or abnormal bone around this region. The patient was managed in a conservative way and was under regular follow up for monitoring of the mass with ultrasound.

*Case* *2:*

**Fig 4:**
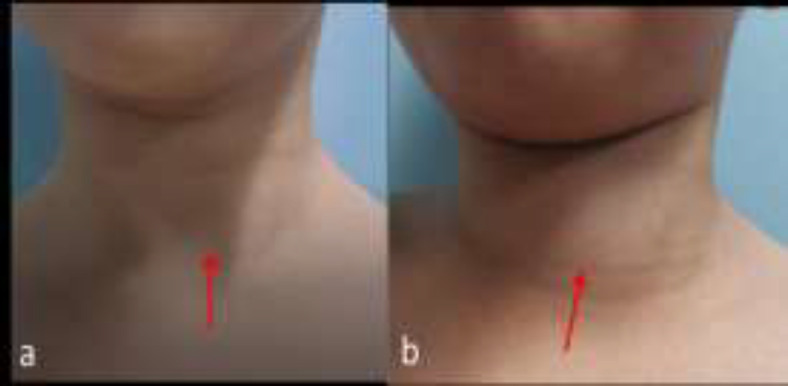
a) There is no visible neck mass over suprasternal region (as shown by red arrow) at rest

A 6-year-old boy was referred to our centre for the assessment of an intermittent neck swelling. His main complaint was apparent neck swelling at central of the neck for 5 months duration in which it appeared only during straining, laughing or shouting. It was painless and no history suggestive of infection or compressive symptoms. His medical history, birth history and family history were unremarkable. Upon neck examination, an anterior neck swelling at the suprasternal region was palpable during the Valsalva maneuver (Fig. 4a,4b). 

b) There is no prominent neck mass over suprasternal region (as shown by red arrow) during Valsalva maneuver.

The swelling was soft in consistency, smooth in surface, not tender and no skin changes. Flexible nasopharyngolaryngoscopy examination was normal. Ultrasound neck showed a well-encapsulated hypoechoic soft tissue mass with scattered speckled echogenic foci seen at the left cervical region inferolateral to left thyroid lobe. It had similar echogenicity as the thymus was and it was separated from the mediastinal thymus. There was mediastinal thymus which demonstrated superior cervical extension above the clavicle to the left cervical region during increased intrathoracic pressure (Fig.5a,5b). 

**Fig 5a:**
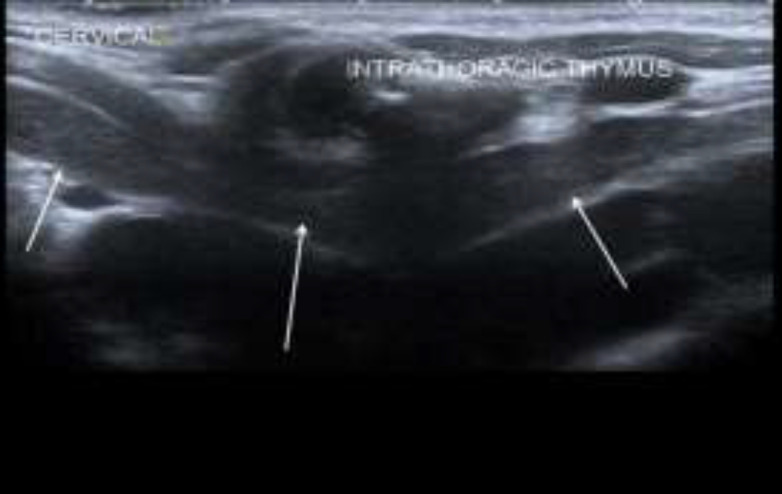
Ultrasound scan taken in sagittal view at left parasternal region shows thymus in the thoracic inlet which is continuous with the intrathoracic thymus (white arrows) when patient is at rest

**Fig 5b:**
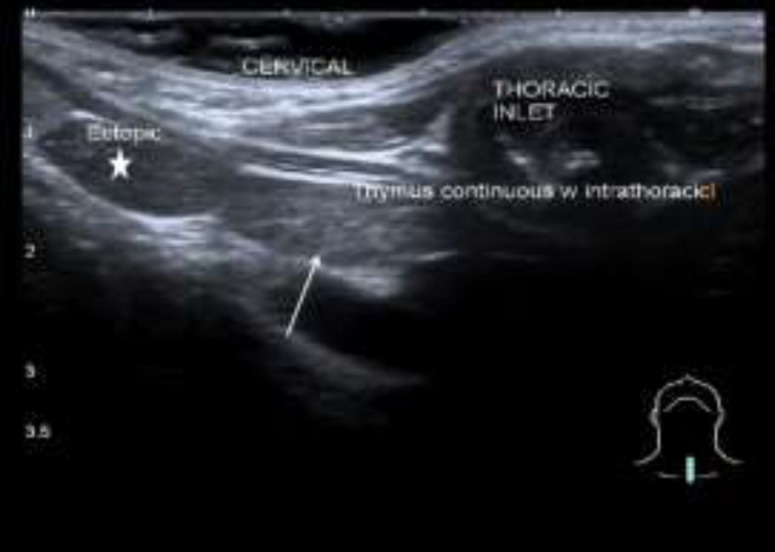
Ultrasound neck in sagittal plane at the level of suprasternal region shows the intrathoracic thymus (white arrows) is continuous with a portion of thymus that dynamically seen slides into suprasternal region upon Valsalva maneuver. A separated ectopic thymus (white star) noted more cranially in the cervical region

Both internal jugular vein and common carotid artery were patent. The thyroid gland was normal in size and echogenicity. 

The decision was made to manage the patient conservatively with regular follow up to monitor the symptoms and size of the mass clinically and also with ultrasound. The patient remained asymptomatic with no progression of the neck mass at 3 month follow-up. 

## Discussion

Embryologically, thymus develops from the third and fourth pharyngeal pouch at the time of sixth gestational week. During the seventh week, the bud-like thymic primordia elongates into thymopharyngeal ducts that migrate caudally and medially to their final location in the anterior mediastinum. Once the thymus reaches its ultimate destination, the thymopharyngeal duct is obliterated (5). Arrest of this embryologic descent can gives rise to a clinical spectrum of thymus anomalies such as ectopic or aberrant thymus. Deposition of the ectopic thymic tissue may occur at any points from the angle of the mandible to the manubrium of the sternum along the descent pathway of the thymus (4). 

According to the literature worldwide, slightly more than 100 cases of cervical ectopic thymus have been described with only 10% occurred in infants. The cases generally occurred between 2 and 15 years of age, with male predominance (7). The most common clinical presentation of the ectopic thymus is painless neck swelling, which was reported in 90% of the cases. Merely 10% of the cases were described to have compressive symptoms such as dyspnea, stridor, and/or swallowing difficulties (5).

This is consistent with our cases in which both patients manifested with painless neck swelling. The interesting point to be discussed is the unusual presentation of the neck swelling in the cases. For the first patient in our series, the 15-month-old girl presented with intermittent midline neck swelling at the suprasternal region during the Valsalva maneuver. It was identified as an ectopic thymus using ultrasound scan of the neck. The superior herniation of the ectopic thymus was rarely described in the previously published cases (5,6)

Generally, aberrant thymic tissue often presented as neck swelling in the lateral aspect of the neck with the preponderance to the right side (4). For the second patient in our study, the 6-year-old boy had similar intermittent suprasternal midline mass with Valsalva maneuver which was identified as superior cervical extension of the normal mediastinal thymus. In addition to that, he also had concomitant left cervical swelling which was diagnosed to be ectopic thymus. Superior cervical extension of the thymus above the manubrium into the lower neck was interpreted as normal anatomy in paediatric age group (2). Despite the normal variant of anatomy, it was infrequently described as intermittent neck mass which was visible only during increased intrathoracic pressure in several case reports (3). Thus far, there is no published case of coexisting superior cervical herniation of normal thymus and ectopic cervical thymic mass in a patient. 

Our hypothesis is that the normal or ectopic thymus may be located in the thoracic inlet at rest while during the Valsalva maneuver the mass was pushed up to the neck with the increased intrathoracic pressure. The presumed theory of herniation of the thymus is due to the loose connective tissues surrounding the thymus that permit the thymic herniation superiorly into the lower neck (1).

Ultrasound is the sole imaging to evaluate the neck masses in this study due to its lack of ionizing radiation, the relatively short examination time,cost-effective and the ability to dynamically assess the mass especially in cases of intermittent mass appearance (1). The characteristic “starry sky” appearance is extremely useful in identifying normal thymus. The variation of the shape of the thymus with cardiac and respiratory movements on realtime ultrasound is also helpful to differentiate it from solid tumors and infiltrative diseases (8). 

Jugular phlebectasia was excluded as the mass was not compressible, and meanwhile, laryngocoele and lung herniation were excluded due to the absence of air visualized in the neck mass (3). The unique sonographic features can confirm the ectopic thymus and hence may not require biopsy or surgical removal for diagnosis confirmation (9). In our present cases, both patients did not proceed with further imaging due to the established sonographic features. 

For the management of the ectopic thymus, it remains controversial. Previously reported cases recommended empiric surgical excision mainly due to the inability to obtain diagnosis through alternative means, concerns of malignant transformation and low recurrence after surgical excision (10). However, cases with thymoma and ectopic thymic carcinoma have been reported in the literature, the occurrence of these tumors are mainly in adults. Furthermore, the literature reviews did not have sufficient data to prove greater malignant transformation rate in the ectopic thymus in comparison to the native thymus (9). 

Surgical excision of thymic neck masses in children results in significant morbidity as the risk of adherence to vital structures such as the jugular vein, carotid artery, and/or the recurrent laryngeal, vagus, hypoglossal and phrenic nerves. Besides that, some cases demonstrated mediastinal connection that possibly needs a sternotomy for total excision. Furthermore, the removal of a cervical thymic mass without the presence of mediastinal thymus can result in immunologic dysfunction (10). 

In addition, the thymus undergoes physiological changes in which it achieves its largest relative size at age of 3 years and attains its largest weight at puberty before involuting in adulthood (5). In view of the natural history of involution of the ectopic thymus, the ‘wait and watch’ approach should be considered as an optimal course of management if the diagnosis was confirmed and if patients did not have compressive symptoms. Medical treatment such as antibiotics or steroid therapy may play a role in thymic hyperplasia following vaccination or related to the infectious process. The limitation is the low incidence of ectopic cervical thymus and hence a prospective randomized study is not feasible (10). 

## Conclusion

Despite its rarity, ectopic thymus should be considered in the list of differential diagnosis of asymptomatic neck masses, specifically in infants and children. 

Location of the lesion along the thymus descent pathway and unique sonographic features make the diagnosis of ectopic cervical thymus easily established. Recognition of the 

atypical presentation of the ectopic thymus as prominent neck swelling only during the Valsalva maneuver is crucial to avoid unnecessary tests and radiation as well as the need of biopsy and surgical intervention. Conservative management with serial ultrasound will be needed by means of longitudinal follow-up.
